# Multiscale Analysis of Functional Surfaces Produced by L-PBF Additive Technology and Titanium Powder Ti6Al4V

**DOI:** 10.3390/ma16083167

**Published:** 2023-04-17

**Authors:** Damian Gogolewski, Paweł Zmarzły, Tomasz Kozior

**Affiliations:** Faculty of Mechatronics and Mechanical Engineering, Kielce University of Technology, al. Tysiąclecia Państwa Polskiego 7, 25-314 Kielce, Poland

**Keywords:** wavelet transformation, multiscale analysis, surface texture, L-PBF, additive manufacturing

## Abstract

The article discusses experimental studies assessing the possibility of mapping surfaces with a characteristic distribution of irregularities. Tests involved surfaces produced using the L-PBF additive technology, using titanium-powder-based material (Ti6Al4V). An evaluation of the resulting surface texture was extended to cover the application of a modern, multiscale analysis, i.e., wavelet transformation. The conducted analysis that involved using selected mother wavelet enabled production process errors and involved determining the size of resulting surface irregularities. The tests provide guidelines and enable a better understanding of the possibility of producing fully functional elements on surfaces, where morphological surface features are distributed in a characteristic way. Conducted statistical studies showed the advantages and disadvantages of the applied solution.

## 1. Introduction

The fourth industrial revolution is a concept that covers the technological and organizational transformation process. Its particularly important aspects are modern manufacturing techniques, especially 3D printing technologies, which enable rapid production of prototypes and models with a complex geometry [1]. The development of additive technologies that allow the production of elements of any complex shape determines the applicability of these methods over an ever-wider spectrum. The production of fully functional components using 3D printing has been implemented in many industrial areas: founding [2], automotive [3], aerospace [4], or pneumatic and hydraulic industries [5], as well as a basis for the production of elements with specific properties or as medicinal aspects [6]. Despite its numerous advantages, these technologies also exhibit disadvantages, which often make the mapping of a CAD-designed model seem problematic. It is particularly evident in the case of free-form and rough, irregular surfaces with specific morphological features. Process limitations, such as minimum layer thickness or aspects of approximating a model with a triangle mesh (most common STL file), translate directly to the quality of produced features and their shapes and sizes [7]. The resulting geometrical surface structure is defined by a number of process parameters (e.g., material, layer thickness, printing direction, laser power and speed for contour and infill parameters, laser beam diameter and path parameters, gas atmosphere, support material placement, further thermal processes, etc.), but also by material parameters including chemical composition and powder parameters like grain distribution and size, which can reach values greater than the layer thickness, depending on the material [8]. A comprehensive analysis of additively manufactured parts also requires an assessment of the potential existence of internal defects in the material [9,10].

The development of modern technologies has also determined the need to research measuring techniques and evaluation methods [11]. It is a common belief that traditional perception and evaluation of a surface structure through Gaussian transformations (roughness or waviness assessment) is insufficient and does not provide a complex of information about morphological surface features [12,13,14,15]. Therefore, new methods were developed [16], as well as hybrid methods that use both classical and multiscale approaches in their data evaluation [17,18]. Multiscale procedures provide a wider spectrum of information on the studied surfaces and enable presenting them on many scales, depending on the type and size of individual surface features [19]. There are currently ongoing studies on the adaptation of multiscale methods for surface texture assessment. Various types of transformations are developed, including sliding bandpass filters, structural functions [20], geometric methods [21,22], or wavelet transformations. Wavelet transformations are used in an increasing number of cases of surface metrology [23,24,25,26,27,28]. The properties of individual wavelets enable an effective and comprehensive assessment of non-periodic irregularities [29], assessment, diagnostics, and indication of the place of occurrence for individual features [30,31], evaluation of manufacturing process parameters [32], tool wear and damage [33], surface texture extraction [34], engineering surface separation [35], or the estimation of surface roughness parameters based on surface images [36].

Based on the current state-of-the-art, it should be concluded that wavelet analysis is an appropriate tool that could be successfully developed to verify the applicability of modern additive technologies in terms of producing characteristic surface features (surface with a characteristic irregularity distribution). It potentially provides great opportunities in terms of measurement signal diagnostics and requires a more in-depth analysis. Please note that there are no studies aimed at evaluating the applicability of wavelet transformations to verify surfaces with a characteristic irregularity distribution for diagnosing the production process, and to assess process errors and irregularity distribution. The previously used, classical filtration methods exhibit limitations and often do not emphasize significant irregularities of components, which are crucial for additive processes. The studies fill the research gap and improve the applicability of modern multiscale methods, which are part of the Fourth Industrial Revolution, Metrology 4.0.

## 2. Materials and Methods

**Figure 1:**
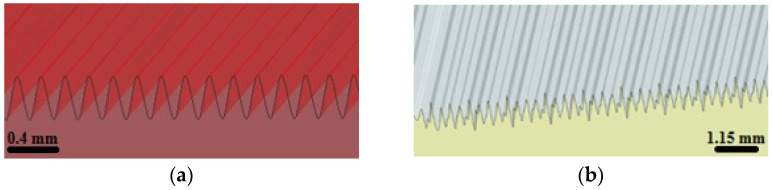
Visualization of produced surfaces (**a**) No. 1–3, (**b**) No. 4–6.

Test samples used to model surfaces with a specific distribution of irregularities were designed in the NX software (Siemens, Plano, TX, USA). Six samples with a surface defined by specific period and amplitude values were executed. Surfaces No. 1–3 were defined using a period function with a period equal to 0.2 mm and an amplitude of 0.34 mm. Surfaces No. 4–6 were defined using a composition of four periodic functions with periods of 0.4; 0.3; 0.25; and 0.2 mm, and amplitudes of 0.5; 0.14; 0.01; and 0.34 mm, respectively. The samples were saved as .stl files using the SolidWorks software (Dassault Systèmes SolidWorks Corp., Waltham, MA, USA), with a linear and angular accuracy of +/− 0.01 mm. Figure 1 shows a visualization of produced surfaces.

**Figure 2:**
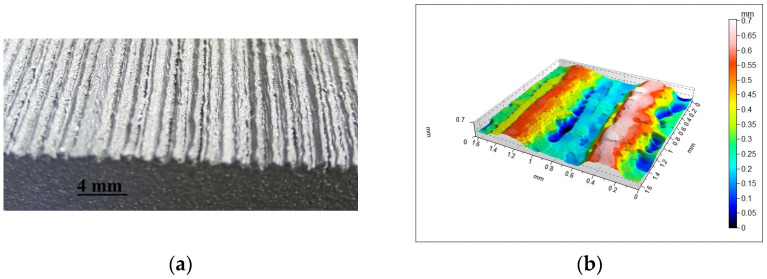
Sample No. 6 (**a**) surface view; (**b**) measured surface isometric image.

Tests involved surfaces produced using the L-PBF additive technology. The samples were made from a titanium-powder-based material (Ti6Al4V), produced by EOS (EOS GmbH, Krailling, Germany) [37]. A 3D printer EOS M290 machine was used to build the sample. Samples No. 1–3 and No. 4–6 were built as an angle increment function relative to the building platform (20°, 45°, 70°). The samples were made with the following technological parameters: Inskin laser power—340 W, laser spot size—100 µm, laser speed—1250 mm/s, hatch distance—0.12 mm, layer thickness—60 µm. The platform temperature was set at a value of 35 °C, argon was used as a shielding, the powder fulfilled ASTM F1472 and ASTM F2924 standards, and samples were heat treated (necessary to stress-relieve treatment) at 800 °C for 2 h in an argon inert atmosphere as instructed by EOS. A surface view of sample No. 6 is shown in Figure 2a.

The measurements of the modelled surface irregularities distribution were conducted using an optical profilometer Talysurf CCI Lite (Taylor Hobson, Leicester, UK) with a vertical resolution of up to 0.01 nm. A magnification equal to ×10 was used for measurements, resulting in a surface size of 1.64 × 1.64 mm, which was represented by a point matrix of 1024 × 1024. TalyMap Platinum 6 (Digital Surf, Besançon, France) and Matlab software (The MathWorks, Natick, MA, USA) were used in the study. An isometric view of the measured sample No. 6 is shown in Figure 2b.

In addition, in order to provide a comprehensive analysis of research samples, the study was enhanced by analysing the samples using SEM (scanning electron microscope) and micro-CT (microfocus computed tomography). Microstructure studies were conducted using a scanning electron microscope JEOL JSM-7100F (JEOL Ltd. Akishima, Tokio, Japan) with different magnifications. The CT scanning and analysis were carried out using a computed tomography system (NIKON M2 LES SYSTEM (Nikon, Minato, Tokio, Japan)) that combines three radiation sources, i.e., two micro- and one minifocus X-ray sources (225 kV, 450 kV, and 450 kV, respectively). The examinations were conducted using a 225 kV X-ray tube with a 2 mm thick copper filter. The scanning data were then processed and visualized using VG Studio 3.5.2. software (Volume Graphics GmbH Heidelberg, Germany). The images were segmented using gray-scale thresholding. The 3D geometry was obtained using a 3 × 3 median filter. In addition, to remove small voids and inclusions, remove options were applied for objects up to 2 voxels in size. Measurements were made with these set parameters: voltage 210 kV, current 195 μA, power 41.0 W, voxel size 30.01 μm, exposure total 1.42 s.

## 3. Results

**Figure 3:**
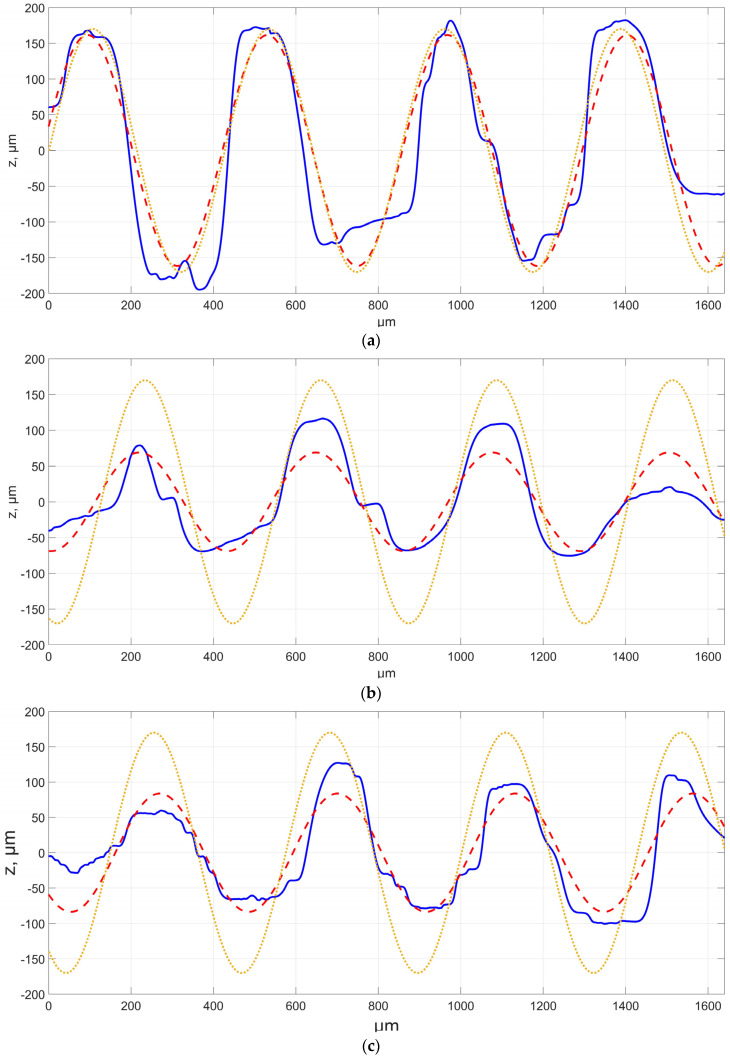
Example of a surface profile for samples No. 1–3 with an approximating function, respectively, as a function of building angle (**a**) 20°, (**b**) 45°, (**c**) 70°. The blue color indicates the measured profile, the red color indicates approximation, and the green color indicates the CAD model.

**Figure 4:**
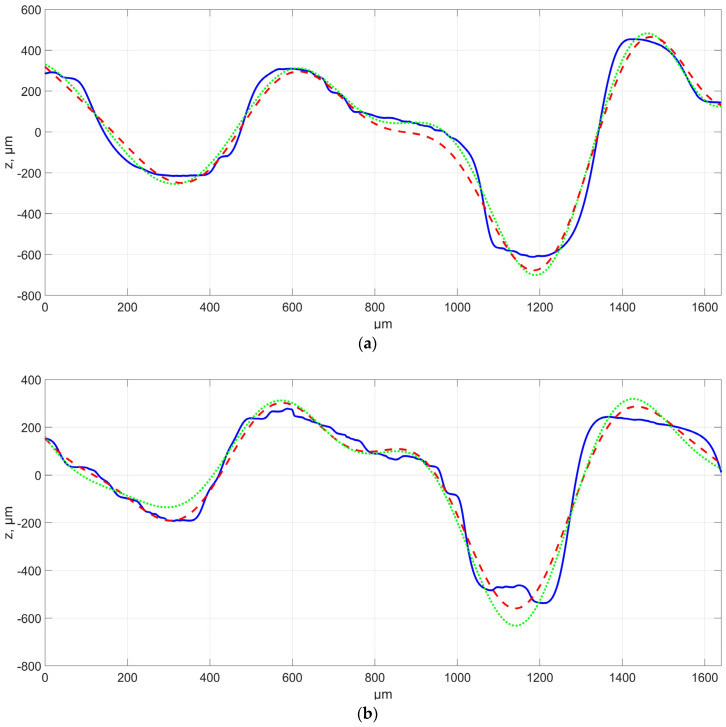

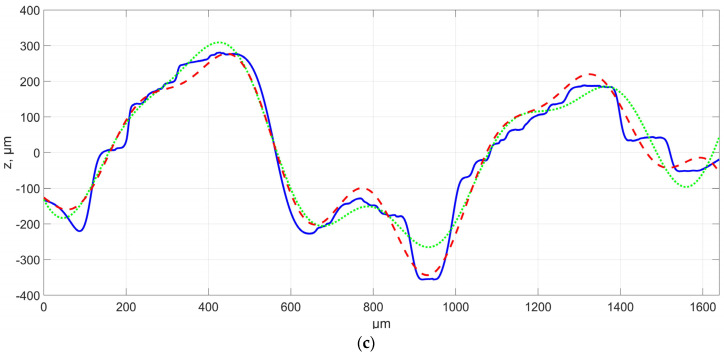
Example of a surface profile for samples No. 4–6 with an approximating function, respectively, as a function of construction angle, (**a**) 20°, (**b**) 45°, (**c**) 70°. The blue color indicates the measured profile, the red color indicates approximation, and the green color indicates CAD model.

The first analysed aspect involved experimental studies focusing on assessing the feasibility of mapping surfaces with a characteristic distribution of irregularities using additive technology. Series with thirty surface profiles perpendicular to the modelled irregularities distribution were assessed for each sample surface produced at a different angle. Successively, each of the surface profiles was approximated, i.e., surfaces No. 1–3 with one periodic function (Figure 3), while for samples No. 4–6, there were four periodic functions (Figure 4). In addition, the modeled CAD function profile is provided in Figure 3 and Figure 4. In the figures below, the abscissa axis shows the measurement section while the ordinate axis shows the height of the irregularity.

Tests showed that the distribution of irregularities on the evaluated surfaces was close to nominal. However, the presence of morphological surface features was recorded due to, among other factors, spreading of the material between individual irregularities or the incomplete formation and melting of individual irregularities. The occurrence intensity of such features was variable and depended on the location on the sample. Approximating surface profiles with periodic functions enabled estimating the possibility of producing a surface of characteristic irregularities distribution. The studies showed that, for samples No. 1–3 defined by one period function, the R^2^ factor for matching the approximating function to the measured, assessed profile ranged from about 0.7 to about 0.85. It should also be noted that the coefficient value decreased as a function of the building angle increasing. The differences may have been caused by, among other factors, difficulties in accurate model mapping and incomplete melting of individual peaks, which can be seen in, e.g., Figure 3b for the end profile. The causes also included limitations to the production process in terms of layer thickness and model approximation, which led to the formation of additional patterns on individual sinusoid waves, for which the height difference corresponded to the assumed layer thickness. It was also noted that the values of the defined approximating function were not fully consistent with the theoretical model. Amplitude values differed relatively by approximately twenty percent on average, depending on the profile. Additionally, in this case, the value decreased when the angle increased. However, it should be noted that periodic function values were convergent with theoretical ones. A relative difference in the values for the assessed profiles was around a few percent. No significant impact of the positioning angle in the case of assessed surface profile was recorded for this parameter.

A similar analysis was conducted for the surfaces of samples 4–6, which were defined by four periodic functions defined by different amplitudes and periods. The R^2^ matching coefficient value for these samples was more than 0.95. Similarly like in the case of surfaces defined by one periodic function, the matching coefficient values decreased together when the building angle increased; however, these changes were insignificant. The studies involved assessing amplitude and period values for each of the four functions. However, please note the presence of one amplitude with a value close to assumed accuracy. It can be presumed that it will not be correctly mapped on the surface; however, it is important to approximate the profile based on four sine curve functions, due to the assumed period of a given function. When analysing the obtained result, it should be concluded that the relative amplitude for the assessed profiles was about thirty percent on average, while the average relative difference of the period value was approximately several percent. For a certain group of surface profiles, the indicated lowest amplitude value was not recorded for approximated functions, which directly translated to the value of other signal amplitudes. It cannot be clearly concluded whether the application of more functions resulted in better or worse mapping of the surface. Analysing the results of surface profile measurements, and taking into account the influence of powder particles on the profile parameters, it can be assumed that the chaotic character of surface irregularities of individual valleys and peaks is determined by the nature of the technological process in which, among other things, there are areas of not fully melted powder. The analysis carried out in relation to the measured and approximated profile showed that the function approximates the tested profile in a very effective way, and the differences between individual points of the profiles are less than 10 μm for the location of the model on the platform at an angle of 20°. For the other angles, the differences reached up to 100 μm. It seems that the location of manufactured models for smaller angles in relation to the building platform allows for the manufacture of a much more precise modeled surface with a noticeably smaller number of technological defects.

**Figure 5:**
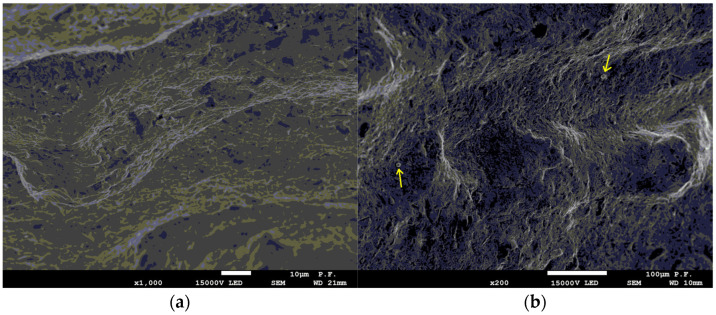

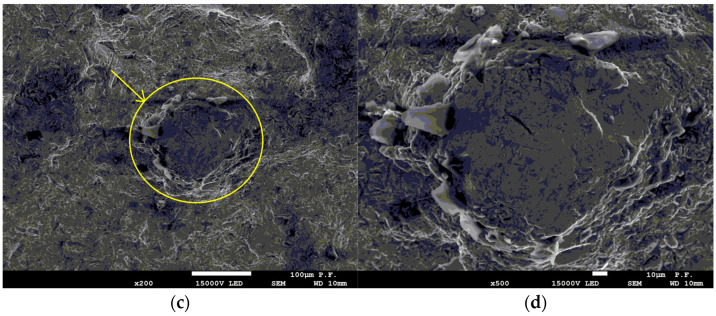
The microstructure of an example test sample: (**a**) surface view, (**b**) minor impurities, (**c**) agglomerated powder grains ×200, (**d**) agglomerated powder grains ×500.

In terms of the resulting errors of the manufacturing process, for the samples in terms of the surface texture based on the analysis carried out using a scanning electron microscope, several characteristic morphological features can be distinguished. No unmelted powder grains were found on the tested surfaces (Figure 5a). Only minor impurities were found, which, due to their frequency of occurrence and their size, cannot be identified as roughness or waviness during optical measurements (please see the arrows in the Figure 5b). In addition, on the surface, there were single areas of melting of agglomerated powder grains (marked area in the Figure 5c,d), but this phenomenon was very rare (found only at 70° samples), such that its influence on the measurement is negligibly small. In this case, irregularities of up to 50–60 μm in height and 100–110 μm in diameter appeared on the surface. Due to the rarity of the discussed technological errors on the tested surfaces and evaluated profiles, the influence of the above features on the profiles of the samples was not noted.

**Figure 6:**
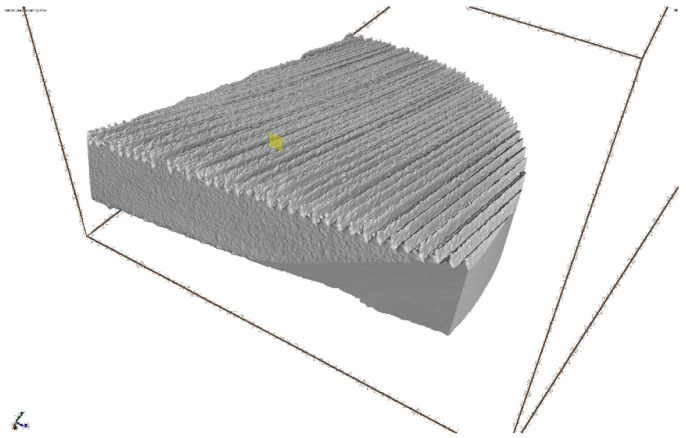
Visualization of the measurement results using CT for the selected sample.

Additional tests conducted using computer tomography showed that the geometric structure of the surface and the material on the analysed cross-sections of all measured profiles did not reveal any material defects related to the technological process of melting metal powders. No cavities, discontinuities, or inclusions were found. Observations near the measured surface layer do not indicate a possible influence of technological defects on the shape of the modeled irregularities. The visualization of the measurement results using CT for the selected sample is presented in Figure 6.

**Figure 7:**
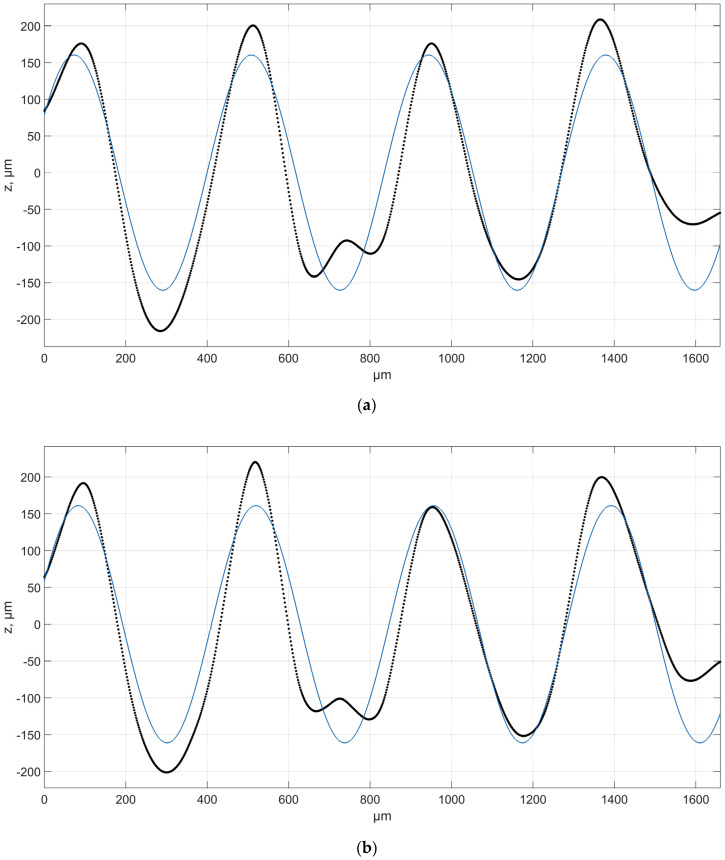

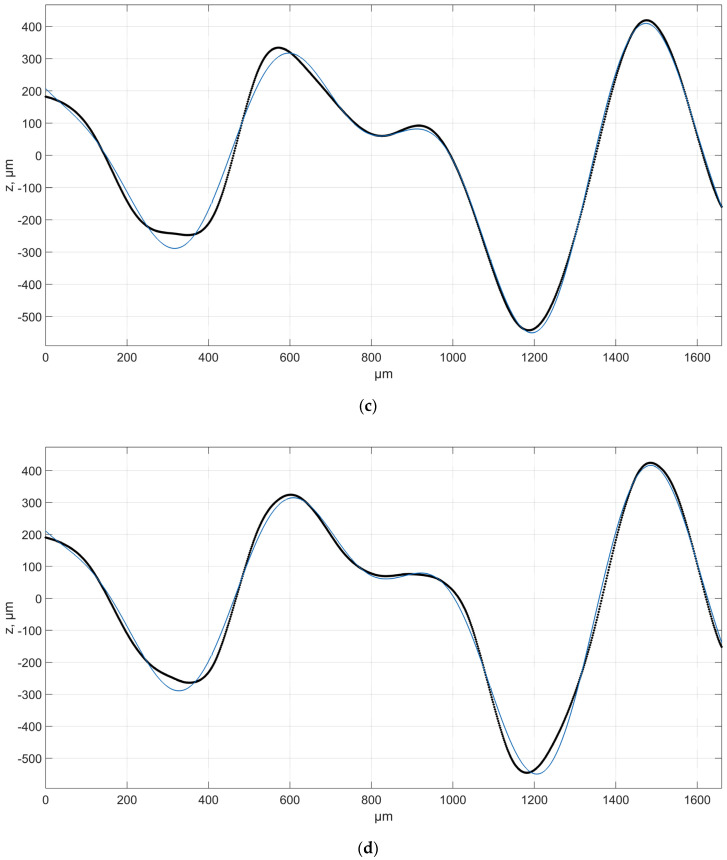
Example of a surface profile together with a sixth analysis level approximation function: (**a**) sample No. 1 coif5 wavelet, (**b**) sample no. 1 sym8 wavelet, (**c**) sample no. 4 coif5 wavelet, (**d**) sample no. 4 sym8 wavelet. The black color indicates the profile obtained by wavelet analysis, the blue color indicates approximation.

Tests focusing on evaluating the possibility of mapping characteristic morphological surface features using additive technologies indicated the validity of comprehensive assessment of the resulting surface texture. Therefore, it was concluded that the application of modern, multiscale analysis, i.e., wavelet transformations, was justified. The research was aimed at assessing the possibility for noise reduction and study production process errors, as well as determining the size of resulting surface irregularities. A one-dimensional, discrete wavelet transformation was used for this purpose. A number of mother wavelet forms with different characteristics and properties were selected for the analysis. The following wavelets were used: db2, db12, db20, coif5, sym2, sym8, bior1.5, bior2.4, bior3.9, bior5.5. Figure 7 shows examples of surface irregularities distribution for samples No. 1 and 4, resulting from the application of coif5 and sym8 mother wavelets at the sixth analysis level. In the figures below, the abscissa axis shows the measurement section while the ordinate axis shows the height of the irregularity.

When assessing the obtained profile, it can be observed that the matching values for the coefficients describing the profile and approximating function were much better than for the profile before filtration. Therefore, it can be inferred that the dominating components of errors in a profile with a regular feature distribution are small, high-frequency pieces of information resulting from production process errors and corresponding to surface pores. Similar tests were conducted for all one hundred and eighty assessed profiles. The studies showed that, for profiles of samples No. 1–3, the authors obtained a matching coefficient value that grew with decomposition progress. However, this tendency can be observed up to the sixth decomposition level. Filtration of further levels for this mother wavelet leads to profile distortion. The tests covered all selected wavelets. Period and amplitude parameter values were greatly dependent on the mother wavelet support length. It was observed that better profile matching coefficients were obtained for mother wavelets with a longer support and that the obtained relative period difference values for individual wavelets were lower than nominal, compared to the parameters determined for the non-filtered profile. Furthermore, signal smoothing and filtering high-frequency information resulted in a change of the approximating function amplitude value. These values improved by several percentage points, depending on the applied wavelet.

Similarly, matching coefficient values for samples 4–6 were determined along with the progress of decomposition. Information that the filtration level leads to profile distortion from the sixth level for this mother wavelet was obtained for surfaces described by the sum of periodic functions. In the case of the indicated level, the obtained values for the studied surfaces were most similar to nominal ones, analogous to samples modelled using a single periodic function. However, for the assessed mother wavelet forms, these values slightly decreased together with wavelet support width increase.

## 4. Discussion

Modern additive technologies enable producing fully functional models. However, the key issue is assessing the possibility of producing complex, characteristic morphological features on the surface of elements, since they directly impact the operation of individual machine parts at a later stage. Research was focused on evaluating the feasibility of producing characteristic irregularities distribution on the surface and process control. The studies were expanded with multiscale assessment of the resulting surface texture, based on discrete, one-dimensional wavelet transformation. The conducted analysis enabled expanding surface diagnostics or process capabilities through a broad and comprehensive assessment of individual surface features. The research provides hints in terms of producing elements, as well as indicates possible process errors, filling the research gap in the field of process diagnostics through assessing additively produced surfaces.

Research carried out using a scanning electron microscope did not indicate the regular, systematic occurrence of unmelted powder grains or impurities. This is a significant advantage over other materials where, in the case of additive technologies, the occurrence of unmelted particles and other defects causing deterioration of surface quality and misinterpretation of test results are observed.

Understanding the manner and scale in which a production process impacted morphological features required a comprehensive evaluation of individual processes, in order to analyse all important geometry aspects that resulted from it. A classic ISO-based perception of surface textures seems to be insufficient in these aspects [38], due to high process complexity [39].

Studies assessing the possibility of producing characteristic surface features indicated high potential applicability of additive technologies. An impact of the building angle on the resulting feature distribution was observed. Both the value of the matching coefficient reduced as a building angle function and the value of parameters describing individual function on the surfaces reached values that differed relatively from nominal values by approximately several percent on average. At the same time, it was noted that increasing the number of functions describing surfaces led to an ambiguous change in the aforementioned parameters.

Multiscale analysis using discrete, one-dimensional wavelet transformations showed dominant surface irregularities components. Assessing irregularities distribution on many scales enabled evaluating the production process in terms of porosity and additional features formed on the surface. The studies showed that filtering out high-frequency components at the initial analysis levels resulted in an improvement of the assessed parameters. Therefore, it can be inferred that the initial differences in the values were caused by random micro-roughness. The research covering a wide spectrum of mother wavelets enabled verifying the impact of a mother wavelet and its properties on the process of filtering individual surface profiles. It also provided hints on the potential diagnostic possibilities associated with the wavelet method.

The conducted tests also came with certain limitations, to be analysed as part of research in the future. In particular, future studies should focus on the greater differentiation of building angles, materials, process parameters like layer thickness or surface types, among others, through adding more functions or analysis free-form surfaces and specified, characteristic locations on individual surfaces. The research will help find functional dependencies of the production process and will translate to its in-depth diagnostics and understanding of the production process for individual morphological features.

## 5. Conclusions

The article assesses the applicability of additive technologies for shaping characteristic irregularities distribution on surfaces. The study involved using a modern approach based on wavelet transformation. An analysis of the results presented above led to the following conclusions:

1. It is possible to manufacture precise models with characteristic morphological features of various sizes and shapes using additive technologies. Based on the scanning electron microscope and computed tomography analysis, it can be noted that there are no defects caused by the technological process and no unmelted powder grains on the tested surfaces. The production of surfaces with a much more complicated shape should not be problematic for additive technologies compared to the limitations known for conventional technologies such as machining. The research has shown that it is a clear advantage compared to conventional methods, where shaping such irregularities and defined morphological features on the surface is hindered or sometimes impossible.
2. It cannot be clearly concluded whether the application of more surface modelling functions resulted in better or worse mapping of the model surface. In the case of surfaces described by a single function (compared approximation function and measured profile), the differences in the parameters differed relatively by an average of approximately twenty percent in terms of the amplitude and several percent in terms of the period, for a matching value of 0.7–0.85, depending on the profile, which means a correlation dependence according to J.P. Guildford’s classification. In the case of a surface defined by several periodic functions, these parameters differed by thirty and several percent, respectively, which for a matching of more than 0.95 proves a very clear correlation dependence. Moreover, comparing the profile specified in the CAD model with the profile of the approximating function, there are differences in the accuracy of the fit depending on the printing direction of the sample models. The most favorable variant due to amplitude and periodic differences is to place the models at the smallest possible angle to the building platform: for the assessed samples, it was an angle equal to 20°. In this case, the amplitude differences reached only a few micrometers. A reduction in the surface irregularities mapping quality was observed with increasing building angle (printing direction), which has a negative effect on the building time, layer number, and stair-step effect.
3. When analysing the data obtained through wavelet filtration, it can be concluded that the dominant error component was high-frequency information resulting from production process errors and corresponding to surface pores. An assessment of the resulting signals leads to a conclusion that signals from the sixth level upwards do not contain such information.
4. In the case of mother wavelets with a large support, the obtained profile-matching coefficients, as well as the approximating function period and amplitude values, were better. However, they slightly decreased when support increased. This tendency could be observed up to the sixth decomposition level. Unnatural distortion of the resulting signals was observed at further levels.
5. The research showed that wavelet transformation can be successfully applied as a diagnostic tool in surface texture assessment and used as a base to diagnose the production process. It seems that a significant limitation of the technological process is that the layer thickness is determined, among other things, by the size of the powder grains, and in future research, it will be possible to analyse much more precisely manufactured models using multiscale analysis.

## Data Availability

The data presented in this study are available upon request from the corresponding author.
